# The Use of Spermina

**Published:** 1893-01-21

**Authors:** 


### THE USE OF SPERMINA.

M. ALEXANDRE PŒHL, in an article in the "Comptes Rendus" on the use of spermina in nitro-organic oxydation, remarks: "Its most remarkable effects are observable in nervous maladies, complicated with anæmia, where we know that the physiological oxydations are enfeebled; neurasthenia, hemiplegia, hystero-epilepsy, and angina pectoris. In fact, the large number of doctors who have used spermina have recorded its tonic effects and a general augmentation of health, which I attribute to increased organic oxydation. If life is a continued struggle against death, spermina is probably one of the most effective agents for the cells of this resistance."

